# Hemostatic radiotherapy in clinically significant tumor-related bleeding: excellent palliative results in a retrospective analysis of 77 patients

**DOI:** 10.1186/s13014-023-02391-5

**Published:** 2023-12-20

**Authors:** Manuel Guhlich, Teresa Esther Maag, Leif Hendrik Dröge, Andrea Hille, Sandra Donath, Stephanie Bendrich, Markus Anton Schirmer, Friedemann Nauck, Martin Leu, Joachim Riggert, Julia Gallwas, Stefan Rieken

**Affiliations:** 1 Clinic of Radiotherapy and Radiation Oncology, University Medical Center Göttingen, Göttingen, Germany; 2 Department of Palliative Medicine, University Medical Center Göttingen, Göttingen, Germany; 3 Department of Transfusion Medicine, University Medical Center Gottingen, Göttingen, Germany; 4 Clinic of Gynecology and Obstetrics, University Medical Center Göttingen, Göttingen, Germany

**Keywords:** Cancer bleeding, Radiotherapy, Palliative therapy, Transfusion, Retrospective study, Emergency radiation

## Abstract

**Background:**

Significant bleeding of tumor sites is a dreaded complication in oncological diseases and often results in clinical emergencies. Besides basic local and interventional procedures, an urgent radiotherapeutic approach can either achieve a bleeding reduction or a bleeding stop in a vast majority of patients. In spite of being used regularly in clinical practice, data reporting results to this therapy approach is still scarce.

**Methods:**

We retrospectively analyzed 77 patients treated for significant tumor-related bleeding at our clinic between 2000 and 2021, evaluating treatment response rate, hemoglobin levels, hemoglobin transfusion necessity, administered radiotherapy dose and overall survival.

**Results:**

Response rate in terms of bleeding stop was 88.3% (68/77) in all patients and 95.2% (60/63) in the subgroup, wherein radiotherapy (RT) was completed as intended. Hemoglobin transfusions decreased during treatment in a further subgroup analysis. Median overall survival (OS) was 3.3 months. Patients with primary tumors (PT) of the cervix (carcinoma of the cervix, CC) or endometrium (endometrioid carcinoma, EDC) and patients receiving the full intended RT dose showed statistically significant better OS in a multivariable cox regression model. Median administered dose was 39 Gy, treatment related acute toxicity was considerably low.

**Conclusions:**

Our data show an excellent response rate with a low toxicity profile when administering urgent radiotherapy for tumor related clinically significant bleeding complications. Nonetheless, treatment decisions should be highly individual due to the low median overall survival of this patient group.

**Supplementary Information:**

The online version contains supplementary material available at 10.1186/s13014-023-02391-5.

## Introduction

Malignant tumor associated bleeding is reported to occur in up to 10% of cancer patients [1]. Tumor bleeding can be caused by local infiltration of blood vessels, tumor angiogenesis or tumor regression due to antineoplastic therapy [2]. Clinically significant tumor bleeding (definition partly based on the ASPREE trial as (1) requirement of red blood cell concentrates (RBCC) or (2) admission to the hospital for > 24 h or prolonged hospitalization with bleeding as the primary reason, [3]) occurs often in advanced tumor stages, when a curative approach is not feasible [4]. In these circumstances, it is of major importance to account for palliative guidance according to the patients’ wishes and needs [5, 6].

If therapy is desired, as will be in most cases, advised local therapies include packing and tamponade, operative or endoscopic cauterization [7] as well as transcutaneous embolization [8]. Often, red blood cells have to be supplemented [9]. Radiation therapy (RT) has been shown to achieve bleeding reduction or bleeding stop in a vast majority of administered patients [4, 10–12]. An enhanced platelet adhesion to the extracellular matrix by an increase of von Willebrand factor was demonstrated in human cells ex vivo to be a possible short-term mode of action [13]. Vascular fibrosis and tumor regression are prolonged (hemostyptic) effects of RT [14]. Due to the difficulty of the clinical setting, the wide variety of primary tumors and multiple possible interfering mechanisms such as anticoagulation and thrombopenia, mostly retrospective data have been published [2, 15]. Prospective studies have been report on gynecological [16, 17] and colorectal [18, 19] malignancies, in respiratory malignancies with more considerable patient numbers [20–27]. Despite several publications concerning the impact of RT on clinically relevant tumor bleeding, the numbers of patients published is still considerably low. In order to broaden the fundamental data concerning hemostatic RT in significant bleeding of various primary cancers, we performed the present retrospective analysis.

## Methods

This single center study retrospectively analyzed patients receiving urgent RT for clinically relevant malignant tumor bleeding. Treatment took place at the Department of Radiotherapy and Radiooncology at the University Medical Center in Göttingen, Germany, between 01/2000 and 06/2021. Patients and their respective diagnoses were identified by systematic keyword screening for “clinically significant bleeding”. Data were extracted from physical patient records and RT treatment planning systems (Varian Eclipse, version 15.6, Varian Medical Systems, Palo Alto, USA). Patient follow-up was evaluated through screening of hospital intern data processing systems (ixserv.4, version R20.3, ix.mid software technology, Köln, Germany) and ONKOSTAR (version 2.9.8, IT-Choice Software AG, Karlsruhe, Germany).

The main study interest was the achievement of symptom relief in terms of a clinically determined bleeding stop. Additionally, the need of ongoing transfusions during the course of RT as well as hemoglobin levels were evaluated. Furthermore, we analyzed overall survival.

Statistical analyses were performed using SPSS (v. 26) and R (v. 4.0.2) with the “KMWin” (Kaplan–Meier for Windows) plugin [28]. Survival data were displayed by Kaplan–Meier plots with statistics for survival time comparisons performed by log-rank tests. Univariable cox regression was applied for assessing impact of variables on survival, univariable logarithmic regression likewise in regard to symptom relief. We considered p-values < 0.05 as statistically significant. Univariably significant variables were consecutively tested in a multivariable fashion.

## Patients

A total of 77 patients were eligible for analysis. Please refer to Flowchart 1 for patient selection.

Patient age ranged from 24 to 89 years (median: 70). Almost 65% (n = 50) of patients were female. Charlson Comorbidity Index (CCI) was ≥ 4 points for 58% of the study population. 32 (41.6%) patients showed clinically significant bleeding as first symptom of their malignant disease, 22 (28.6%) patients had recurrent disease. Primary tumors were predominantly pelvic gynecological malignancies (CC; n = 19, ENC; n = 9), and non-small cell lung cancer (NSCLC; n = 13). Applied RT dose ranged from 9 to 84.4 Gy (median: 39 Gy). 30 patients had received chemotherapy of any kind prior to the current bleeding event. Intended RT course could be completed in 63 (81.8%) patients. Fourteen (18.2%) patients were aborted during therapy, including five (6.5%) patients that died during the emergency RT course. In nine (11.6%) patients, treatment was adjusted to a curative radio(chemo)therapy concept after palliation was successful. 68 patients were considered to be in a palliative state initially and throughout RT due to recurrent disease or due to metastases. RT was very well tolerated with acute treatment related side effects not exceeding Grade 2 according to CTCAE V5.0 [29]. For patient-, disease- and treatment characteristics please refer to Table 1, 2, 3, 4 and 5.

**Table 1:** Patient, disease and treatment characteristics

Patients, N (%)	77
Age (years), median (min–max)	70 (24–89)
Sex: female:male, N (%)	50 (64.9):27 (35.1)
*Charlson comorbidity index*	
1–3	32 (41.5)
4–6	30 (39)
7–10	15 (19.5)
*Disease characteristics*	
Bleeding as first symptom of disease	32 (41.6)
Recurrent disease, N (%)	22 (28.2)
*Treatment characteristics*	
Dose, median (min–max)	39.0 Gy (9–84.4)
Chemotherapy, prior to acute bleeding symptomatic (N (%))	30 (39.0)
Immunotherapy, any (N (%))	2 (2.6)

**Table 2:** Tumor entities assigned by anatomical region

Tumor entities: N (%)	
Pelvic malignancies	46 (59.7)
Carcinoma of the cervix	19 (24.7)
Endometrioid carcinoma	9 (11.7)
Prostate carcinoma	4 (5.2)
Bladder carcinoma	3 (3.9)
Rectum carcinoma	3 (3.9)
Ureter carcinoma	3 (3.9)
Ovarial carcinoma	2 (2.6)
Uterus sarcoma	1 (1.3)
Anal carcinoma	1 (1.3)
Pelvic CUP	1 (1.3)
Thoracic malignancies	19 (24.7)
Non-small cell lung cancer	13 (16.9)
Breast carcinoma	3 (3.9)
Esophageal carcinoma	1 (1.3)
Small cell lung cancer	1 (1.3)
Abdominal malignancies	7 (9.1)
Colon carcinoma	2 (2.6)
Renal cell carcinoma	3 (3.9)
Gastric carcinoma	1 (1.3)
Liposarcoma	1 (1.3)
Pancreatic carcinoma	1 (1.3)
Head and neck malignancies	3 (3.9)
Hypopharynx carcinoma	1 (1.3)
Carcinoma of the tongue	1 (1.3)
Oropharynx carcinoma	1 (1.3)
Skin cancer	2 (2.6)
Malignant melanoma	2 (2.6)

**Table 3:** Radiotherapy treatment details

Course of radiotherapy (RT): N (%)	
Intended RT completed	63 (81.8)
Intended RT incomplete	14 (18.2)
Death during RT	5 (6.5)
Symptom relief: all patients	68 (88.3)
Symptom relief: patients with intended RT complete	60 (95.2)
Change to curative concept	9 (11.7)
RT technique: N (%)	
3D conformal RT (3DcRT)	55 (71.4)
Volumetric modulated arc therapy (VMAT)	14 (18.2)
Intensity modulated RT (IMRT)	3 (3.9)
Brachytherapy	3 (3.9)
3DcRT + VMAT	2 (2.6)
3DcRT + IMRT	1 (1.3)

**Table 4:** Acute treatment related side effects according to CTCAE V5.0 [29]

Acute treatment-related side effects (CTCAE V5.0)		
Acute side effects, any: N (%)	35 (45.5)	
	Grade 1	Grade 2
Skin erythema	13 (16.9)	4 (5.2)
Esophagitis	1 (1.3)	1 (1.3)
Emesis	6 (7.8)	–
Cystitis	5 (6.5)	2 (2.6)
Enteritis	13 (16.9)	1 (1.3)
Proctitis	9 (11.7)	3 (3.9)

**Table 5:** Details concerning applied RT dose and fractionating scheme for all patients of the study (N = 77) with corresponding EQD_2_ (α/β:10) and BED_10_

Applied Dose (Gy)	1st Fractionation (Fractions*Gy)	2nd Fractionation (Fractions*Gy)	3rd Fractionation (Fractions*Gy)	EQD_2_ (α/β:10)	BED_10_	Patients: N (%)	Primary Tumor	Bleeding stop achieved (%)	Comments
9	3 × 3			9.75	11.7	4 (5.6)	H&N, pelvic A-CUP, CC, LC	50	2/4 RT aborted due to death of patient
10	1 × 10			16.67	20	1 (1.3)	OC	100	Brachytherapy
10	2 × 5			12.5	15	1 (1.3)	BC	100	RT aborted prematurely after achieving bleeding stop due to general deterioration
12	4 × 3			13	15.6	2 (2.6)	CC, EC	100	
12	6 × 2			12	14.4	1 (1.3)	CC	100	RT aborted prematurely after achieving bleeding stop due to patients’ decision
13	3 × 3	2 × 2		13.75	16.5	1 (1.3)	pelvic A-CUP	0	RT aborted prematurely due to patients’ decision before achieving bleeding stop
18	3 × 3	5 × 1.8		18.6	22.32	1 (1.3)	PC	100	RT aborted prematurely due to general deterioration
19	3 × 3	5 × 2		19.75	23.7	1 (1.3)	EDC	0	1/14 RT aborted prematurely without achieving bleeding stop due to patients ‘ decision
20	5 × 4			23.33	28	1 (1.3)	RCC	100	
20	4 × 5			25	30	1 (1.3)	RC	100	
40	5 × 8			60	72	1 (1.3)	UC	100	
30	6 × 5			37.5	45	1 (1.3)	H&N	100	Brachytherapy
24	1 × 3	1 × 5	1 × 4	27.17	32.6	1 (1.3)	EDC	100	4th Fractionation: 4 × 3 Gy
24	4 × 2	4 × 4		26.67	32	1 (1.3)	LC	0	RT aborted prematurely
28.8	3 × 3	11 × 1.8		29.22	35.06	1 (1.3)	CC	100	RT aborted prematurely due to general deterioration
30	15 × 2			30	36	3 (3.9)	MM: 2, CC	100	1/3 low dose due to Reirraditation
30	10 × 3			32.5	39	4 (5.2)	CC, BC, EC, PC	50	4/4 RT aborted prematurely, 2/4 due to complications, 1/4 due to death by pulmonary artery embolism, 1/4 after achieving bleeding stop due to patients’ decision
30.6	3 × 3	2 × 1.8	9 × 2	31.29	37.55	1 (1.3)	CC	100	
30.6	3 × 3	12 × 1.8		30.99	37.19	1 (1.3)	BLC	100	
33	11 × 3			35.75	42.9	1 (1.3)	LC	0	RT aborted due to death of patient
36	13 × 2			36	43.2	1 (1.3)	BLC	0	RT aborted due to death of patient
36	12 × 3			39	46.8	2 (2.6)	EDT	100	1/2 RT aborted prematurely after achieving bleeding stop due to patients’ decision
39	13 × 3			42.25	50.7	14 (18.2)	EDT: 3, LC: 3, UC: 3, BLC: 2, PC, H&N, RC	85.7	
40	20 × 2			40	48	4 (5.2)	GC, CC; BLC, PC	100	
45	15 × 3			48.75	58.5	2 (2.6)	BLC	100	
45	3 × 3	18 × 2		46.45	54.9	3 (3.9)	CC, EDT, US, UC	100	
45	1 × 3	21 × 2		45.25	54.3	1 (1.3)	EDT	100	
45	3 × 3	20 × 1.8		45.15	54.18	2 (2.6)	CC, OC	100	
45	25 × 1.8			44.25	53.1	4 (5.2)	CC, RC, AC, PaC	100	
49	3 × 3	20 × 2		49.75	59.7	2 (2.6)	CC, BLC	100	
50	25 × 2			50	60	1 (1.3)	LS, RC	100	
50.4	28 × 1.8			49.56	59.47	1 (1.3)	BLC	100	Change to curative concept after achieving bleeding stop
54	3 × 3	25 × 1.8		54	64.8	1 (1.3)	RC	100	Change to curative concept after achieving bleeding stop
59	3 × 3	25 × 2		59.75	71.7	1 (1.3)	LC	100	Change to curative concept after achieving bleeding stop
59.4	33 × 1.8			58.41	70.09	1 (1.3)	CC	100	
59.4	3 × 3	28 × 1.8		59.31	71.17	7 (9)	CC	100	5/7 Change to curative concept after achieving bleeding stop, additional Brachytherapy
65	5 × 3	25 × 2		66.25	79.5	1 (1.3)	LC	100	Change to curative concept after achieving bleeding stop

*CC* carcinoma of the cervix, *BC* breast carcinoma, *BLC* bladder cancer, *EC* esophageal carcinoma, *EDC* endometrioid carcinoma, *OC* ovarial carcinoma, *LC* lung cancer, *A-CUP* Adeno-Cancer of unknown primary, *H&N* cancer of the head and neck, *RC* rectum carcinoma, *PC* prostate carcinoma, *RCC* renal cell carninoma, *AC* anal carcinoma, *GC* gastric carcinoma, *LS* liposarcoma, *PaC* pancreatic carcinoma, *MM* malignant melanoma, *UC* urothel carcinoma other than bladder, *US* uterine sarcoma

If not stated otherwise in the comment section, percutaneous RT was applied

## Results

**Fig. 1:**
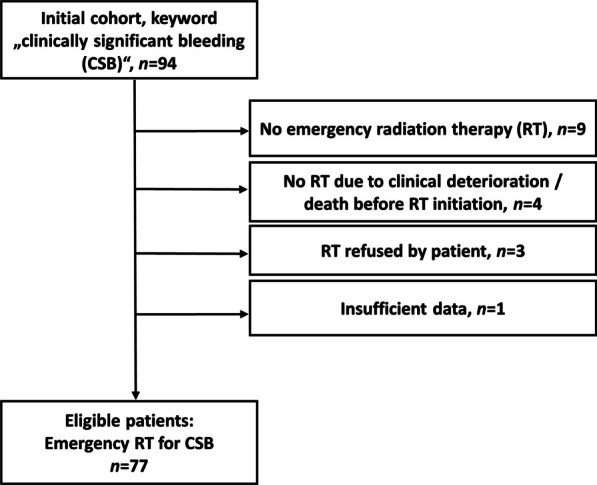
Flowchart of patient selection. Screening of keyword “clinically significant bleeding” was performed from 01/2000 to 06/2021

**Fig. 2:**
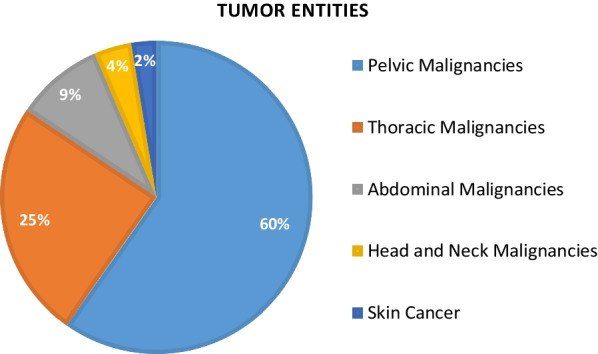
Pie chart: distribution of patients primary tumors divided by anatomical regions. For details concerning primary tumors, please refer to Table 2

Bleeding remission, defined as a clinically determined bleeding stop during RT, was achieved in 88.3% of patients (n = 68). Regarding patients that completed the intended RT regime (n = 63), 95% (n = 60) reached this endpoint. Table 6 comprises details of potential influencers for a successful bleeding stop. In a univariable logistic regression, CCI, applied dose in Gy and completion of therapy as intended were statistically significant. When tested multivariable, completion of the intended therapy remained statistically significant (Figs. 1 and 2).

**Table 6:** Influence of potential prognostic factors on patients’ bleeding stop

Variable (n)	Symptom relief: clinically determined bleeding stop		
	Hazard ratio (95% CI)	*P* value univariable	*P* value multivariable
Age	1.03 (0.98–1.08)	0.27	
Sex			
Male (27) versus female (50)	0.28 (0.06–1.28)	1.01	
CCI	0.67 (0.46–0.99)	**0.04**	n.s
Dose in Gy	1.07 (1.01–1.13)	**0.02**	n.s
Transfusion(s) necessary			
Yes (30) versus No (47)	2.05 (0.39–10.89)	0.40	
Bleeding as first sign of disease			
Yes (32) versus No (45)	1.21 (0.27–5.45)	0.81	
Systemic therapy			
Yes (30) versus No (47)	0.88 (0.51–1.52)	0.64	
Acute organ toxicity			
Yes (35) versus No (42)	0.69 (0.41–1.19)	0.18	
Localization pelvis versus other			
Yes (46) versus No (31)	2.29 (0.52–10.02)	0.27	n.s
Therapy completed as intended			
No (14) versus Yes (63)	0.44 (0.01–0.26)	** < 0.01**	**0.01**
Radiotherapy technique^a^			
Dynamic (17) versus conventional (55)	0.57 (0.12–2.63)	0.47	

Calculations were done by logistic regression analyses. P values < 0.05 were considered statistically significant. Variables with p < 0.1 in univariable analysis were consecutively tested in a multivariable logaritmic regression model

*CI* confidence interval, *CCI* Charlson Comorbidity Index, *Gy* Gray, *n.s.* not significant

^a^Not applicable in n = 6 patients (n = 3 brachytherapy, n = 3 mixed techniques, Table 3)

Statistically significant values (*P*<0.05) are depicted in bold

**Fig. 3:**
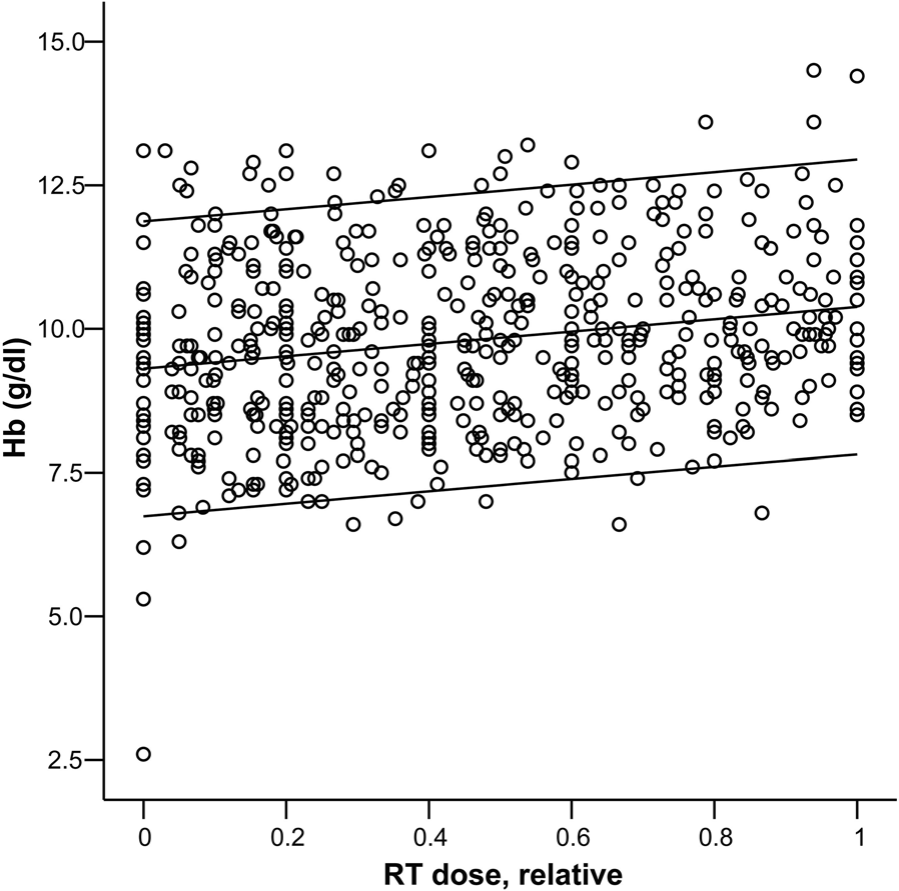
Hemoglobin levels (Hb, grams/deciliter, y-axis) of patients with at least two documented data points during emergency radiation therapy for clinically significant tumor bleeding (n = 76). X-axis: relative RT dose (completed percentage of the RT-series) applied. Each dot represents one Hb-level of one patient during RT at a specific relative administered RT dose. Lines indicating 95% confidence interval and median

**Fig. 4:**
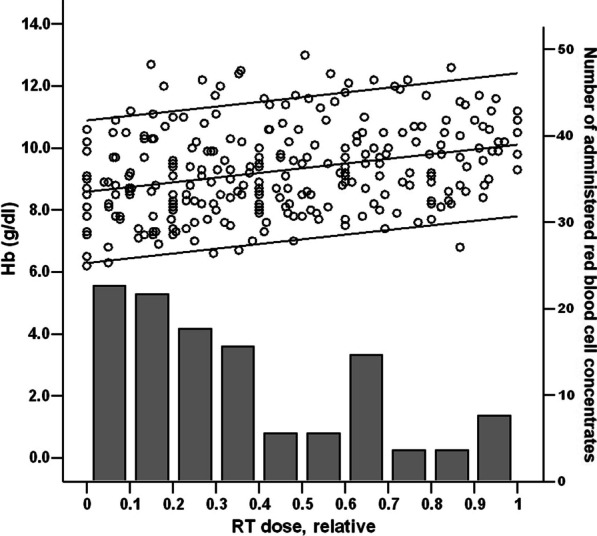
Combination of hemoglobin levels (Hb, grams/deciliter, Y-axis left) as depicted in Fig. 3 with columns representing combined absolute red blood cell transfusions during RT course (Y-axis right). X-axis: relative RT dose (completed percentage of the RT-series) applied. Data available for n = 27 patients

Besides clinical evaluation of bleeding remission, patients’ blood cell counts (BCC) were monitored during therapy. For n = 76 patients (98.7%), two or more BCC were documented and evaluated. Please refer to Fig. 3 for a depiction of these 76 patients, indicating rising hemoglobin levels during the course of RT. To evaluate the hemostyptic effect of RT, we assessed the numbers of transfused RBCC during the RT course. This data was accessible for n = 27 patients (35.1%, Fig. 4).

**Fig. 5:**
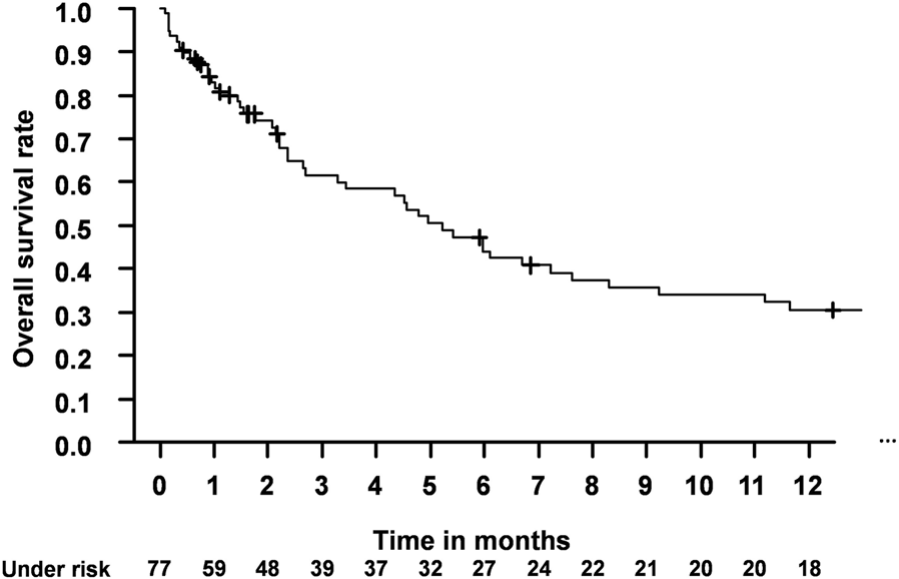
Kaplan Meier estimate for OS

Analyzing survival, median OS was 3.3 months. Please refer to Fig. 5 for Kaplan Meier estimates concerning OS detailing the first 12 months. For a complete Kaplan Meier estimate, please refer to the supplementary material (Additional file 1: Fig. S1).

When evaluating OS in our cohort, female sex, CCI below the cohorts’ median, pelvic primary tumor, combined CC/ENC PT as well as completion of the intended RT dose showed to be influential in a univariable cox regression. When tested multivariable, CC/ENC PT and completion of the intended RT dose remained statistically significant. Please refer to Table 7 for details.

**Table 7:** Influence of potential prognostic factors on patients’ OS

Variable (n)	Overall survival		
	Hazard ratio (95% CI)	*P* value univariable	*P* value multivariable
Age	1.00 (0.98–1.02)	0.97	
≥ 70 (41) versus < 70 (36)	0.77 (0.45–1.30)	0.32	
Sex			
Male (27) versus female (50)	2.26 (1.31–3.90)	** < 0.01**	n.s
CCI			
> 4 (38) versus ≤ 4 (39)	2.02 (1.17–3.45)	**0.01**	n.s
Dose in Gy			
≥ 39 (47) versus < 39 (30)	0.63 (0.37–1.07)	0.09	
Transfusion(s) necessary			
Yes (30) versus No (47)	1.20 (0.70–2.07)	0.51	
Bleeding as first sign of disease			
Yes (32) versus No (45)	0.66 (0.38–1.13)	0.13	
Systemic therapy			
Yes (30) versus No (47)	0.88 (0.51–1.52)	0.64	
Acute organ toxicity			
Yes (35) versus No (42)	0.69 (0.41–1.19)	0.18	
Localization pelvis versus other			
Yes (46) versus No (31)	0.54 (0.32–0.93)	**0.03**	n.s
Therapy completed as intended			
No (14) versus Yes (63)	4.27 (2.07–8.78)	** < 0.01**	** < 0.001**
Radiotherapy technique^a^			
Dynamic (17) versus conventional (55)	0.63 (0.33–1.21)	1.66	
Primary site			
CC/ENC (29) versus others (48)	0.29 (0.16–0.54)	** < 0.01**	** < 0.001**

Calculations were done by cox regression analyses. P values < 0.05 were considered statistically significant. Variables with p < 0.1 in univariable analysis were consecutively tested in a multivariable cox regression model

*CC* carcinoma of the cervic, *ENC* endometrioid carcinoma, *CI* confidence interval, *CCI* Charlson Comorbidity Index, *Gy* Gray, *n.s.* not significant

^a^Not applicable in n = 6 patients (n = 3 brachytherapy, n = 3 mixed techniques, Table 3)

Statistically significant values (*P*<0.05) are depicted in bold

## Discussion

We herein report 77 cases of clinically relevant tumor bleeding treated by radiotherapy in an emergency therapy approach, achieving the determined therapy aim of “bleeding stop” in 88% of administered patients. Literature concerning the efficacy and safety of urgent RT for bleeding tumors is mostly limited to retrospective data and has recently been summarized in a systematic review [15]. Publications concern either cumulative cohorts or specific primary tumor sites, in the latter containing relatively small patient numbers. As far as *cumulative cohorts* are concerned, Cihoric et al. report on a bleeding improvement in 87% (n = 54) of patients and complete bleeding control in 63% (n = 39) of patients [4]. Sapienza et al. [30] documented 89% bleeding control (n = 89), Kumar et al. [11] report 76% (n = 53), Nomoto et al. [31] 83% (n = 15).

In data analyzing *specific tumor sites*, Shuja et al. report 57% (n = 24) of patients reaching complete bleeding control and 31% (n = 13) partial response in a cohort of malignant pelvic tumors [32]. Lacarrière et al. [12] and Tey et al. [33] analyzed RT for hematuria in bladder-cancer; Zhang et al. [34] for urothelial cancer, documenting freedom of hematuria at the end of RT in 69% (n = 28), 76% (n = 39) and 88% (n = 22), respectively. In a prospective pilot study evaluating hemostatic RT for gastric cancer, Tanaka et al. [35] report 80% initial response rate (n = 25) and further 20% (n = 6) to reirradiation. Yu et al. [36], Kondoh et al. [37] and Lee et al. [38] evaluated RT for gastric cancer related bleedings retrospectively, reporting an efficacy of 89% (n = 54) and 73% (n = 11) at the end, and 75% (n = 43) one month after completion of palliative RT, respectively.

Concerning the administered dose and fractionating schedule, Katano et al. [10] report of higher bleeding remission in a group of patients with different primary tumors receiving biologically efficient dose (BED)_10_-equivalent of 39 Gy compared to patients < 39 Gy BED_10_ (91% vs. 71%, not reaching statistical significance, likely due to few patient numbers [n = 36]). Ogita et al. [39] demonstrated a statistically significant effect of BED_10_ ≥ 36 Gy in patients receiving palliative RT for gross hematuria. Tanaka et al. [35] show a significant better OS for higher dose regimes compared to single fraction RT in a prospective pilot study. In our cohort, the mean applied dose (39 at 3 Gy/fraction) reaches a BED_10_ of 50.7 Gy. Even though we did not find a statistically significant effect of the administered dose on OS, these comparably high BED_10_-doses likely have an influence on the excellent clinical bleeding remissions reported. This interpretation is supported by our finding of a hazard ratio of 1.07, when evaluation the administered RT dose in Gray in terms of a bleeding stop. There was no influence of applied RT dose on OS in both of the studies, whereas Cihoric et al. [4] report a significantly better OS in patients’ receiving > 30 Gy compared to < 30 Gy. Butala et al. [40] report in a recent retrospective data series of 33 patients suffering from bleeding complications by pelvic gynecological malignancies, indicating that short-course RT (herein defined as less than or equal to five fractions, > 3.5 Gy/fraction) is equally effective as conventionally fractionated three courses > 5 fractions. Keeping the short median OS of this patient’s cohort in mind, we acknowledge the need to evaluate on a highly individual level for the best of the patients’ needs. Preliminary ending of a palliative treatment as soon as the primary palliative goal is achieved should always be discussed on a day-to-day basis. As these individually tailored approaches regularly are difficult, these discussions should most effectively take place in an experienced interdisciplinary team. This includes experienced palliative care physicians and radiation oncologists and also appears highly useful on an educational level, involving young professionals and possibly even advanced medical students [41–43].

Assessing our presented data, certain limitations have to be addressed. First and foremost, due to the retrospective design, uncontrollable bias might affect our interpretations. Furthermore, we were not able to report a graduation of initial bleeding (e.g., the World Health Organization bleeding scale [44] as well as bleeding remission besides the above mentioned. There is also a lack of consistent follow-up in terms of hemoglobin levels and duration of bleeding remission. Finally, Eastern Cooperative Oncology Group (ECOG) status can not be reported. We do, on the other hand, report on a relatively large patient cohort of excellent symptom control in a clinically relevant emergency setting in oncology. We present comprehensive data verifying rising hemoglobin levels during emergency RT as well as a decreasing need for RBCC transfusion in a well-documented subgroup. Our data furthermore show a small subgroup of patients initially presenting with an acute life-threatening symptom, that received curative RT concepts after achieving the primary goal of bleeding control, resulting in long term survival (n = 8 at 60 months follow-up, Additional file 1: Fig. S1). We therefore broaden the current literature by adding the aforementioned results, helping in finding individually tailored therapy concepts in everyday emergency RT indications.

## Conclusion

In this retrospective analysis, we present data of a large cohort of patients receiving urgent RT for significant tumor-related bleeding. RT was documented to be highly effective in achieving a clinically determined bleeding stop while causing no toxicities exceeding CTCAE II°. Besides rising hemoglobin levels, a decreasing demand for RBCC could be demonstrated in a subgroup analysis. Furthermore, we demonstrate a subgroup of patients that was able to achieve long-term survival despite starting treatment in an emergency setting.

In clinical emergency settings, individually tailored concepts are exceptionally important, respecting the patients’ wishes as well as medically determined needs. For these situations, our data add relevant background information, helping to assess potentially life-saving treatment decisions.

### Supplementary Information

**Additional file 1. **Kaplan Meier Estimate for OS.

## Data Availability

Not applicable.

## Abbreviations

AC: Anal carcinoma
A-CUP: Adeno-carcinoma of unknown primary
BC: Breast cancer
BCC: Blood cell counts
BLC: Bladder cancer
CI: Confidence interval
CC: Carcinoma of the cervix
CCI: Charlson comorbidity index
CTCAE: Common Terminology Criteria for Adverse Evens
ECOG: Eastern Cooperative Oncology Group
EC: Esophageal carcinoma
EDC: Endometroidal carcinoma
GC: Gastric carcinoma
Gy: Gray
H&N: Cancer of the head and neck
LC: Lung cancer
LS: Liposarcoma
MM: Malignant melanoma
n.s.: Not significant
NSCLC: Non-small cell lung cancer
OC: Ovarial carcinoma
OS: Overall survival
PaC: Pancreatic carcinoma
PT: Primary tumors
PC: Prostate carcinoma
RC: Rectum carcinoma
RCC: Renal cell carcinoma
RBCC: Red blood cell concentrates
RT: Radiotherapy, radiation therapy
UC: Urothel carcinoma other than bladder
US: Uterine sarcoma
