# Supplementary figures and images for: Hemostatic radiotherapy in clinically significant tumor-related bleeding: excellent palliative results in a retrospective analysis of 77 patients
Source: Radiat Oncol. 2023 Dec 20;18:203. doi: 10.1186/s13014-023-02391-5 (PMC10734078; doi:10.1186/s13014-023-02391-5)

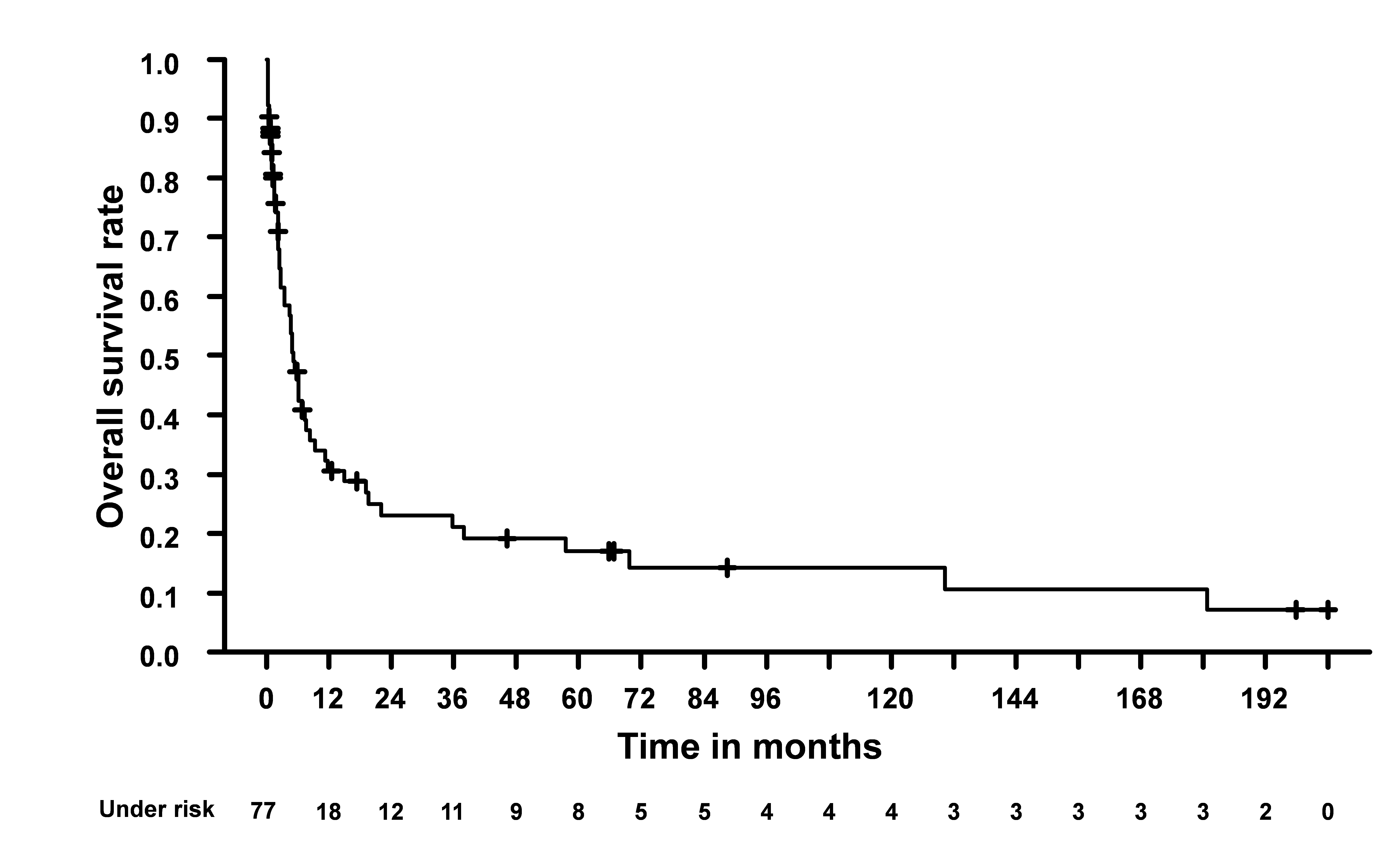

Supplement: Supplementary file 1 — Additional file 1. Kaplan Meier Estimate for OS. [file 13014_2023_2391_MOESM1_ESM.jpg]
